# Supplementary material for: Microbial Phenolic Metabolites: Which Molecules Actually Have an Effect on Human Health?
Source: Nutrients. 2019 Nov 10;11(11):2725. doi: 10.3390/nu11112725 (PMC6893422; doi:10.3390/nu11112725)
Supplement: Supplementary file 1 [file nutrients-11-02725-s001.pdf]

Table S1. Principal MPM found in biological fluids after feeding trials in humans.

GUT MICROBIOTA METABOLITES	FOOD SOURCE	SAMPLE TYPE	REFERENCES
	**BENZOATES**		
**Methyl 4-hydroxybenzoate**	Red raspberry	Breast milk, plasma, urine	[24]
**Methyl-3,4-dihydroxybenzoate**	Red raspberry	Breast milk, plasma, urine	[24]
	**HYDROXYBENZALDEHYDES**		
**4-Hydroxybenzaldehyde**	Red raspberry	Breast milk, plasma, urine	[24]
**4-Hydroxybenzaldehyde glucuronide**	Red raspberry	Urine	[24]
**3,4-Dihydroxybenzaldehyde**	Red raspberry	Breast milk, plasma, urine	[24]
	**HYDROXYBENZOIC ACIDS**		
**Benzoic acid sulphate**	Orange juice	Urine	[26,28]
**3-Hydroxybenzoic acid**	Capsule of epicatechin, cocoa, coffee, grape extract, red wine, tea, tomato sauce	Faeces, plasma, urine	[35,42,43,45,50,51,71,73]
3-Hydroxybenzoic acid sulphate	Orange juice	Urine	[26,28]
3-Hydroxybenzoic acid glucuronide	Orange juice, red raspberry	Breast milk, urine	[24,28]
**4-Hydroxybenzoic acid**	Capsule of epicatechin and procyanidin B1, cocoa, grape extract, orange juice, red raspberry, red wine, tomato sauce	Breast milk, faeces, plasma, urine	[20,24,27,28,42,43,45,50,71,73]
4-Hydroxybenzoic acid glucuronide	Red raspberry	Breast milk, urine	[24]
4-Hydroxybenzoic acid sulphate	Orange juice, red raspberry	Breast milk, urine	[24,28]
**2,3-Dihydroxybenzoic acid**	Red raspberry	Breast milk, plasma, urine	[24]
**2,4-Dihydroxybenzoic acid**	Red raspberry	Urine	[24]
**2,6-Dihydroxybenzoic acid**	Red raspberry	Breast milk, plasma, urine	[24]
**3,4-Dihydroxybenzoic acid**	Coffee, grape extract, orange juice, red raspberry	Breast milk, plasma, urine	[24,28,35,71]
3,4-Dihydroxybenzoic acid sulphate	Red raspberry	Breast milk, urine	[24]
**3,5-Dihydroxybenzoic acid**	Red wine	Faeces	[50,70]

**3-Methoxy-4-hydroxybenzoic acid**	Coffee, orange juice	Plasma, urine	[28,35]
**3-Hydroxy-4-methoxybenzoic acid**	Orange juice	Urine	[28]
Hydroxy-dimethyoxybenzoic acid glucuronide	Grape extract	Urine	[65]
**Gallic acid**	Grape extract, pomegranate extract, red raspberry, red wine	Breast milk, colon tissues, faeces, plasma, urine	[24,50,54,66]
Gallic acid glucuronide	Tea	Blister fluids, skin biopsies	[51]
Gallic acid sulphate	Tea	Plasma	[51]
**3-Methylgallic acid**	Grape extract, red wine	Faeces, urine	[50,66,70]
**4-Methylgallic acid sulphate**	Grape extract, tea	Blister fluids, plasma, skin biopsies, urine	[51,66]
**3,4-Dimethylgallic acid**	Grape extract	Urine	[66]
**Vanillic acid**	Capsule of epicatechin and procyanidin B1, cocoa, red raspberry, red wine	Breast milk, faeces, plasma, urine	[24,42,43,45,50,70]
Vanillic acid sulphate	Grape extract, red raspberry	Breast milk, urine	[24,66]
Vanillic acid glucuronide	Almond skin, grape extract, red raspberry	Breast milk, plasma, urine	[24,71,65]
**Isovanillic acid**	Red raspberry	Breast milk, urine	[24]
Isovanillic acid glucuronide	Red raspberry	Breast milk, plasma, urine	[24]
Isovanillic acid sulphate	Red raspberry	Breast milk, urine	[24]
**Protocatechuic acid**	Capsule of epicatechin and procyanidin B1, cocoa, red wine	Faeces, plasma, urine	[42,43,45,50,70]
**Syringic acid**	Grape extract, red wine	Urine, faeces	[20,50,66,70]
Syringic acid sulphate	Tea	Blister fluids, plasma	[51]
Syringic acid glucuronide	Tea	Blister fluids	[51]
**Ellagic acid**	Pomegranate extract	Colon tissues	[54]
**Methyl-ellagic acid**	Pomegranate extract	Colon tissues, urine	[54]
Methyl-ellagic acid glucuronide	Pomegranate extract	Urine	[54]
**Dimethyl-ellagic acid**	Pomegranate extract	Colon tissues, urine	[54]
Dimethyl-ellagic acid glucuronide	Pomegranate extract	Colon tissues, urine	[54]
**Valoneic acid dilactone**	Pomegranate extract	Colon tissues	[54]
**Gallagic acid dilactone**	Pomegranate extract	Colon tissues	[54]

**Vanilloylglycine**	Grape extract	Urine	[65]
	**HYDROXYCINNAMIC ACIDS**		
***m*-Coumaric acid**	Capsule of epicatechin and procyanidin B1, cocoa, red raspberry, tomato sauce, orange juice	Breast milk, plasma, urine	[24,28,43,45,70]
***p*-Coumaric acid**	Capsule of epicatechin and procyanidin B1, cocoa, grape extract, red raspberry, red wine, tomato sauce, orange juice	Breast milk, faeces, plasma, urine	[20,24, 28,42,43,45,5066, 70]
Coumaric acid glucuronide	Coffee, orange juice, red raspberry, tomato sauce	Breast milk, plasma, urine	[24,28,35,70]
Coumaric acid sulphate	Coffee, orange juice, red raspberry, tomato sauce	Breast milk, plasma, urine	[24,26,28,35,70]
**Dihydrocoumaric acid**	Coffee	Urine	[35]
Dihydrocoumaric acid glucuronide	Coffee	Urine	[35]
Dihydrocoumaric acid sulphate	Coffee	Urine	[35]
**Coumaroylquinic acid**	Coffee	Urine	[35]
**Dihydrocoumaroylquinic acid**	Coffee	Plasma, urine	[35]
**Caffeic acid**	Cocoa, coffee, grape extract, orange juice, red raspberry, red wine, tomato sauce	Faeces, plasma, urine	[20,24,28,35,42,45,50,66,70]
Caffeic acid glucuronide	Orange juice, tomato sauce	Urine	[28,70]
Caffeic acid sulphate	Coffee, grape extract, orange juice, red raspberry, tomato sauce	Breast milk, plasma, urine	[24,26,28,35,66,70]
**Dihydrocaffeic acid**	Coffee, tomato sauce	Plasma, urine	[35,70]
Dihydrocaffeic acid glucuronide	Coffee, grape extract, tomato sauce	Urine	[35,66,70]
Dihydrocaffeic acid sulphate	Coffee, red raspberry, tomato sauce	Urine, breast milk, plasma	[24,35,70]
**3-Caffeoylquinic acid**	Coffee	Urine	[35]
**5-Caffeoylquinic acid**	Coffee, tomato sauce	Plasma, urine	[35,70]
**4-Caffeoylquinic acid**	Coffee	Urine	[35]
Caffeoylquinic lactone sulphate	Coffee	Urine	[35]
**3-Dihydrocaffeoylquinic acid**	Coffee	Urine	[35]
**5-Dihydrocaffeoylquinic acid**	Coffee	Urine	[35]
**4-Dihydrocaffeoylquinic acid**	Coffee	Urine	[35]
Dihydrocaffeoylquinic acid glucuronide	Coffee	Urine	[35]

**Dimethoxycinnamic acid**	Coffee	Plasma	[35]
**Dihydrodimethoxycinnamic acid**	Coffee	Plasma, urine	[35]
**Ferulic acid**	Capsule of epicatechin and procyanidin B1, cocoa, coffee, orange juice, red raspberry, red wine, tomato sauce	Breast milk, faeces, plasma, urine	[20,24,26,28,35,42,43,45,50,70]
Ferulic acid glucuronide	Almond skin, coffee, orange juice, red raspberry, tomato sauce	Breast milk, plasma, urine	[24,26,28,35,70,71]
Ferulic acid sulphate	Coffee, grape extract, orange juice, red raspberry, tomato sauce	Breast milk, plasma, urine	[24,26,28,35,66,70]
**Hydroferulic acid**	Tomato sauce	Urine	[70]
Hydroferulic acid glucuronide	Tomato sauce	Urine	[70]
Hydroferulic acid sulphate	Tomato sauce	Urine	[70]
**Dihydroferulic acid**	Coffee	Plasma, urine	[70]
Dihydroferulic acid glucuronide	Coffee, grape extract, red raspberry	Breast milk, plasma, urine	[24,35,66]
Dihydroferulic acid sulphate	Coffee, grape extract, red raspberry	Breast milk, plasma, urine	[24,35,66]
**Feruloylglycine**	Coffee, red wine	Plasma, urine	[20,35]
**3-Feruloylquinic acid**	Coffee	Urine	[35]
**5-Feruloylquinic acid**	Coffee	Plasma, urine	[35]
**4-Feruloylquinic acid**	Coffee	Plasma, urine	[35]
Feruloylquinic lactone glucuronide	Coffee	Plasma	[35]
**Isoferulic acid**	Coffee, orange juice	Plasma, urine	[28,35]
Isoferulic acid glucuronide	Coffee, orange juice	Urine	[26,28,35]
Isoferulic acid sulphate	Coffee, grape extract, orange juice	Urine	[28,35,66]
**Dihydroisoferulic acid**	Coffee	Plasma	[35]
Dihydroisoferulic acid glucuronide	Coffee	Plasma, urine	[35]
Dihydroisoferulic acid sulphate	Coffee	Plasma, urine	[35]
3-Dihydroferuloylquinic acid	Coffee	Plasma, urine	[35]
5-Dihydroferuloylquinic acid	Coffee	Plasma, urine	[35]
4-Dihydroferuloylquinic acid	Coffee	Urine	[35]
**Isoferuloylglycine**	Coffee	Urine	[35]
	**HYDROXYCOUMARINS**		

**Urolithin A**	Pomegranate extract, red raspberry, strawberry	Breast milk, colon tissues, plasma, urine	[24,53,54]
Urolithin A glucuronide	Pomegranate extract, red raspberry, strawberry	Breast milk, colon tissues, plasma, urine	[24,53,54]
Urolithin A sulphate	Pomegranate extract	Colon tissues, plasma, urine	[54]
Urolithin A sulphate glucuronide	Pomegranate extract	Plasma, urine	[54]
**Isourolithin A**	Pomegranate extract	Colon tissues, plasma, urine	[54]
Isourolithin A glucuronide	Pomegranate extract	Colon tissues, plasma, urine	[54]
**Urolithin B**	Pomegranate extract, strawberry	Colon tissues, plasma, urine	[53,54]
Urolithin B glucuronide	Pomegranate extract, red raspberry, strawberry	Breast milk, colon tissues, plasma, urine	[24,53,54]
Urolithin B sulphate	Pomegranate extract	Colon tissues, plasma, urine	[54]
**Urolithin C**	Pomegranate extract	Colon tissues, urine	[54]
Urolithin C glucuronide	Pomegranate extract	Plasma, urine	[54]
Urolithin C sulphate	Pomegranate extract	Plasma, urine	[54]
**Urolithin D**	Pomegranate extract	Colon tissues, plasma	[54]
**Urolithin M5**	Pomegranate extract	Colon tissues	[54]
**Urolithin M6**	Pomegranate extract	Colon tissues	[54]
	**HYDROXYPHENYLACETIC ACIDS**		
**Phenylacetic acid**	Cocoa, orange juice, red wine, tomato sauce	Faeces, plasma, urine	[26,42,45,50,70]
Phenylacetic acid glucuronide	Tomato sauce	Urine	[70]
Phenylacetic acid sulphate	Tomato sauce	Urine	[70]
**2-Hydroxyphenylacetic acid**	Grape extract, red raspberry	Breast milk, plasma, urine	[24,66]
2-Hydroxyphenylacetic acid sulphate	Almond skin	Urine	[71]
**2-Hydroxy-2-phenylacetic acid**	Grape extract	Urine	[66]
2-(Dihydroxyphenyl)-acetic acid glucuronide	Almond skin	Urine	[71]

2-(Dihydroxyphenyl)-acetic acid sulphate	Almond skin	Urine	[71]
2-(Dihydroxyphenyl)-acetic acid sulphate glucuronide	Almond skin	Urine	[71]
2-(Hydroxy-methoxy-phenyl)-acetic acid glucuronide	Almond skin	Urine	[71]
**3-Hydroxyphenylacetic acid**	Capsule of epicatechin and procyanidin B1, cocoa, coffee, grape extract, orange juice, red raspberry, red wine, tomato sauce	Breast milk, faeces, plasma, urine	[24,26,73,28,35,42,43,45,50,66,70]
3-Hydroxyphenylacetic acid sulphate	Capsule of epicatechin and procyanidin B1, orange juice, tomato sauce	Plasma, urine	[26,28,43,70]
Hydroxyphenylacetic acid glucuronide	Orange juice, tomato sauce	Urine	[28,70]
**4-Hydroxyphenylacetic acid**	Capsule of epicatechin and procyanidin B1, grape extract, orange juice, red raspberry, red wine, tomato sauce	Breast milk, faeces, plasma, urine	[24,26–28,43,50,66,70]
Hydroxyphenylacetic acid sulphate	Orange juice, tomato sauce	Urine	[28,70]
**3,4-Dihydroxyphenylacetic acid**	Capsule of epicatechin and procyanidin B1, cocoa, coffee, grape extract, orange juice, red raspberry, red wine, tomato sauce	Breast milk, faeces, plasma, urine	[20,24,26,28,35,42,43,45,66,70]
**3-Methoxy-4-hydroxyphenylacetic acid**	Cocoa, coffee, orange juice	Urine	[26–28,35,42,45]
Methoxyphenylacetic acid glucuronide	Orange juice	Urine	[26,28]
3′-Methoxyphenylacetic acid sulphate	Orange juice	Urine	[26,28]
**4-Methoxy-3-hydroxyphenylacetic acid**	Coffee	Urine	[35]
4′-Methoxyphenylacetic acid sulphate	Orange juice	Urine	[26,28]
**Homovanillic acid**	Grape extract, red raspberry, red wine, tomato sauce	Breast milk, plasma, urine	[20,24,66,70]
Homovanillic acid sulphate	Grape extract, tea	Plasma, urine	[48,66]
**Isohomovanillic acid**	Red raspberry	Breast milk, plasma, urine	[24]
**3′-Methoxy-4′-hydroxymandelic acid**	Orange juice	Urine	[26,28]
**4′-Hydroxymandelic acid**	Orange juice	Urine	[26,28]
**HYDROXYPHENYLPENTANOIC ACIDS**			
**4-Hydroxy-5-(phenyl)-valeric acid**	Red wine	Faeces	[50,67,68]
4-Hydroxy-5-(phenyl)-valeric acid sulphate	Almond skin	Urine	[71]
**4-Hydroxy-5-(hydroxyphenyl)-valeric acid**	Red wine	Faeces	[50,67,68]

4-Hydroxy-5-(hydroxyphenyl)-valeric acid glucuronide	Almond skin	Urine	[71]
4-Hydroxy-5-(hydroxyphenyl)-valeric acid sulphate	Almond skin	Urine	[71]
4-Hydroxy-5-(hydroxyphenyl)-valeric acid methyl sulphate	Tea	Plasma	[48]
4-Hydroxy-5-(methoxyphenyl)-valeric acid glucuronide	Almond skin	Urine	[71]
4-Hydroxy-5-(hydroxy-methoxyphenyl)-valeric acid glucuronide	Almond skin	Urine	[71]
**4-Hydroxy-5-(3′,4′-dihydroxyphenyl)-valeric acid**	Capsule of epicatechin, dark chocolate, grape extract, red wine	Faeces, plasma, urine	[43,44,50,66,68]
4-Hydroxy-5-(3′,4′-dihydroxyphenyl)-valeric acid glucuronide	Almond skin, dark chocolate, grape extract, tea	Plasma, urine	[44,48,65,71]
4-Hydroxy-5-(3′,4′-dihydroxyphenyl)-valeric acid methyl glucuronide	Dark chocolate	Urine	[44]
4-Hydroxy-5-(3′,4′-dihydroxyphenyl)-valeric acid sulphate	Almond skin, dark chocolate, grape extract, tea	Plasma, urine	[44,48,65,71]
4-Hydroxy-5-(3′,4′-dihydroxyphenyl)-valeric acid methyl sulphate	Dark chocolate, tea	Plasma, urine	[44,48]
4-Hydroxy-5-(3′,5′-dihydroxyphenyl)-valeric acid glucuronide	Tea	Plasma	[48]
4-Hydroxy-5-(3′,5′-dihydroxyphenyl)-valeric acid methyl glucuronide	Tea	Plasma	[48]
4-Hydroxy-5-(3′,4′,5′-trihydroxyphenyl)-valeric acid methyl glucuronide	Tea	Plasma	[48]
**HYDROXYPHENYLPROPANOIC ACIDS**			
**3-Phenylpropionic acid**	Orange juice, red wine	Faeces, urine	[26,50]
3-(Phenyl)propionic acid glucuronide	Orange juice	Urine	[28]
3-(Phenyl)propionic acid sulphate	Orange juice	Urine	[28]
**3-Hydroxyphenylpropionic acid**	Capsule of epicatechin and procyanidin B1, cocoa, coffee, red raspberry, tomato sauce	Breast milk, plasma, urine	[24,35,42,43,45,70]

3-Hydroxyphenylpropionic acid glucuronide	Almond skin, tomato sauce	Urine	[70,71]
3-Hydroxyphenylpropionic acid sulphate	Red raspberry, tomato sauce	Breast milk, urine	[24,70]
**3-(3′-Hydroxyphenyl)propionic acid**	Orange juice, red wine	Faeces, urine	[28,50,67,68]
3-(3′-Hydroxyphenyl)propionic acid glucuronide	Orange juice	Urine	[28]
3-(3′-Hydroxyphenyl)propionic acid sulphate	Orange juice	Urine	[28]
**3-(3′-Hydroxy-4′-methoxyphenyl)propionic acid**	Orange juice	Urine	[27,28]
3-(4′-Methoxyphenyl)propionic acid glucuronide	Orange juice	Urine	[26,28]
3-(4′-Methoxyphenyl)propionic acid sulphate	Orange juice	Urine	[26,28]
**3-(4′-Hydroxyphenyl)propionic acid**	Grape extract, orange juice	Urine	[28,66]
3-(4′-Hydroxyphenyl)propionic acid glucuronide	Orange juice, tomato sauce	Urine	[28,70]
3-(4′-Hydroxyphenyl)propionic acid sulphate	Orange juice, tomato sauce	Urine	[26,28,70]
**3-(3′-Methoxy-4′-hydroxyphenyl)propionic acid**	Orange juice	Urine	[26–28]
3-(3′-Methoxyphenyl)propionic acid glucuronide	Orange juice	Urine	[26,28]
3-(3′-Methoxyphenyl)propionic acid sulphate	Orange juice	Urine	[26,28]
**3,4-Dihydroxyphenylpropionic acid**	Capsule of epicatechin and procyanidin B1, cocoa, grape extract, red wine	Faeces, plasma, urine	[42,43,45,50,66]
**4-Hydroxyphenylpropionic acid**	Capsule of epicatechin and procyanidin B1, cocoa, tomato sauce	Faeces, plasma, urine	[42,43,70]
2S-1-(3-4-Dihydroxyphenyl)-3-(2-4-6-trihydroxyphenyl)-propan-2-ol methyl sulphate	Dark chocolate	Urine	[44]

3-(Dihydroxyphenyl)-propionic acid sulphate	Almond skin	Urine	[71]
Dihydrosinapic acid glucuronide	Grape extract	Urine	[65]
**3-(3′-Hydroxy-4′-methoxyphenyl)hydracrylic acid**	Orange juice	Urine	[26–28]
**3-(3′-Hydroxyphenyl)hydracrylic acid**	Orange juice	Urine	[26–28]
**Hippuric acid**	Cocoa, dark chocolate, grape extract, orange juice, red raspberry, tea	Blister fluids, breast milk, plasma, urine	[24,26–28,42,48,51,66]
Hippuric acid glucuronide	Orange juice	Urine	[28]
**4-Hydroxyhippuric acid**	Almond skin, cocoa, coffee, grape extract, orange juice, tomato sauce	Plasma, urine	[26–28,35,42,45,65, 66,70, 71]
**3-Hydroxyhippuric acid**	Almond skin, cocoa, coffee, grape extract, orange juice, tea, tomato sauce	Plasma, urine	[20,26–28,35,42,45,48,70,71]
**VALEROLACTONES**			
**5-(3′-Hydroxyphenyl)-γ-valerolactone**	Red wine	Faeces	[68]
5-(3′-Hydroxyphenyl)-γ-valerolactone sulphate	Almond skin, dark chocolate, tea	Plasma, urine	[25,44,48,71]
5-(3′-Hydroxyphenyl)-γ-valerolactone glucuronide	Almond skin, dark chocolate, tea	Plasma, urine	[25,44,48,71]
5-(3´-Methoxy,4´-hydroxyphenyl)-γ-valerolactone glucuronide	Cocoa, dark chocolate	Breast milk, plasma, urine	[45,46]
5-(3´-Methoxy,4´-hydroxyphenyl)-γ-valerolactone sulphate	Cocoa, dark chocolate	Breast milk, urine	[45,46]
5-(Hydroxy-methoxy-phenyl)-γ-valerolactone glucuronide	Almond skin, grape extract	Urine	[65,71]
5-(Hydroxy-methoxy-phenyl)-γ-valerolactone sulphate	Almond skin	Urine	[71]
**5-(3′,4′-Dihydroxyphenyl)-γ-valerolactone**	Capsule of epicatechin and procyanidin B1, cocoa, dark chocolate, grape extract, red wine, tea	Breast milk, faeces, plasma, urine, ileostomy fluids	[43–45,49,66-68]

5-(3′,4′-Dihydroxyphenyl)-γ-valerolactone methyl	Capsule of epicatechin	Plasma	[43]
5-(3′,4′-Dihydroxyphenyl)-γ-valerolactone methyl glucuronide	Dark chocolate	Urine	[44]
5-(3′,4′-Dihydroxyphenyl)-γ-valerolactone methyl sulphate	Dark chocolate	Urine	[44]
5-(3′,4′-Dihydroxyphenyl)-γ-valerolactone glucuronide	Almond skin, cocoa, dark chocolate, grape extract, red raspberry	Breast milk, plasma, urine	[24,44–46,65,71]
5-(3′,4′-Dihydroxyphenyl)-γ-valerolactone sulphate	Almond skin, cocoa, dark chocolate, red raspberry	Breast milk, plasma, urine	[24,44–46,71]
5-(3′,5′-Dihydroxyphenyl)-γ-valerolactone glucuronide	Tea	Plasma, urine	[25,48,51]
5-(3′,5′-Dihydroxyphenyl)-γ-valerolactone methyl glucuronide	Tea	Plasma	[48]
5-(3′,5′-Dihydroxyphenyl)-γ-valerolactone sulphate	Tea	Plasma, urine	[25,48,51]
5-(3′,5′-Dihydroxyphenyl)-γ-valerolactone methyl sulphate	Tea	Plasma	[48]
5-(3′,5′-Dihydroxyphenyl)-γ-valerolactone disulphate	Tea	Urine	[25]
5-(4′,5′-Dihydroxyphenyl)-γ-valerolactone glucuronide	Tea	Plasma, urine	[25,51]
5-(4′,5′-Dihydroxyphenyl)-γ-valerolactone sulphate	Tea	Plasma	[51]
5-(4′,5′-Dihydroxyphenyl)-γ-valerolactone sulphate glucuronide	Almond skin, tea	Plasma,urine	[25,48,71]
5-(4′,5′-Dihydroxyphenyl)-γ-valerolactone disulphate	Tea	Urine	[25]
5-(4′,5′-Dihydroxyphenyl)-γ-valerolactone methyl glucuronide	Tea	Urine	[25]
**5-(3′,4′,5′-trihydroxyphenyl)-γ-valerolactone**	Tea	Ileostomy fluids, urine	[25,49]

5-(3′,4′,5′-trihydroxyphenyl)-γ-valerolactone glucuronide	Tea	Plasma, urine	[25,48,51]
5-(3′,4′,5′-trihydroxyphenyl)-γ-valerolactone methyl glucuronide	Tea	Plasma, urine	[25,48]
5-(3′,4′,5′-trihydroxyphenyl)-γ-valerolactone sulphate	Red raspberry, tea	Breast milk, plasma, urine	[24,25,48,51]
5-(3′,4′,5′-trihydroxyphenyl)-γ-valerolactone methyl sulphate	Tea	Plasma, urine	[25,48,51]
5-(3′,4′,5′-trihydroxyphenyl)-γ-valerolactone sulphate glucuronide	Tea	Plasma	[48]
5-(4′-trihydroxyphenyl)-γ-valerolactone glucuronide	Almond skin, tea	Plasma, urine	[48,71]
	**OTHERS**		
**Phloroglucinol**	Coffee, orange juice, red raspberry	Plasma, urine	[24,26,28,35]
Phloroglucinaldehyde	Red raspberry	Plasma	[24]
Catechol (1,2-dihydroxybenzene) methyl sulphate	Tea	Plasma	[48]
**Pyrogallol**	Grape extract, tea	Ileostomy fluids, urine	[49,66]
Pyrogallol(1,2,3-trihydroxy-benzene) sulphate	Tea	Plasma	[48]
Pyrogallol(1,2,3-trihydroxy-benzene) glucuronide	Tea	Plasma	[48]
2-Methylpyrogallol	Grape extract	Urine	[66]
Lunularin	Grapevine-shoot supplement	Urine	[19]
